# The dynamics of trkA expression in the bovine ovary are associated with a luteotrophic effect of ovulation-inducing factor/nerve growth factor (OIF/NGF)

**DOI:** 10.1186/s12958-016-0182-9

**Published:** 2016-08-20

**Authors:** Rodrigo Carrasco, Jaswant Singh, Gregg P. Adams

**Affiliations:** Department of Veterinary Biomedical Sciences, Western College of Veterinary Medicine, University of Saskatchewan, 52 Campus Drive, Saskatoon, S7N 5B4 Canada

**Keywords:** Bovine, Ovary, Corpus luteum, Tyrosine kinase A receptor, Ovulation-inducing factor, Nerve growth factor

## Abstract

**Background:**

Ovulation-inducing factor in semen (OIF/NGF) influences ovulation and CL form and function in camelids and, remarkably, in cows. To test the hypothesis that the luteotrophic effect of OIF/NGF is mediated by an increase in trkA receptors in the ovulatory follicle and early CL, a study was designed to characterize the spatial and temporal distribution of trkA in ovarian follicles and CL at known stages of the bovine estrous cycle.

**Methods:**

Sexually mature cattle (*n* = 14) were examined daily by transrectal ultrasonography to determine the day of ovulation (Day 0), and assigned randomly to be unilaterally ovariectomized on Day 2, 4, 6 or in the pre-ovulatory period just before or after exogenous LH treatment. After a complete interovulatory interval, the cows were re-assigned to a different day–group on which the remaining ovary was removed. Sections of ovarian tissue representing the dominant follicle, largest subordinate follicle, and the CL were processed for immunofluorescent detection and quantification of trkA receptor.

**Results:**

TrkA immuno-fluorescence in ovarian tissues was restricted to follicles and the CL (no reaction in stroma or vessels), and was restricted to the cytoplasm (no nuclear staining). The trkA staining intensity, area of staining, and proportion of cells stained was greater in both theca and granulosa layers of dominant follicles than in that of subordinate follicles (*P* ≤ 0.05) in all day-groups except the Pre-LH group. Among dominant follicles, a progressive reduction in the immuno-positive reaction was detected from Day 2 to Day 6. Among subordinate follicles, immuno-reactivity remained low and unchanged except a rise in the Pre-LH group. The number of immuno-positive cells was greater in early developing CL (Days 2 and 4 combined) than in mature or regressing stage CL (Day 6, Pre- and Post-LH combined; *P* = 0.01). The intracellular distribution of trkA was more diffuse and widespread in dominant than subordinate follicles, particularly on Day 2 and Post-LH (*P* < 0.05).

**Conclusions:**

Distinct differences in trkA expression between dominant and subordinate follicles, particularly when circulating progesterone is minimal (early luteal development and after luteolysis) is consistent with a local role of OIF/NGF in follicle selection and early luteogenesis.

## Background

Ovulation-inducing factor (OIF) is a protein in the seminal plasma that elicits an ovulatory response in camelids when administered intramuscularly, intravenously or by intrauterine infusion [1–4]. The protein has subsequently been identified as beta nerve growth factor (NGF [5]), and is present in the seminal plasma of all species examined to-date [6]. The existence and effect of this seminal protein challenge the classic assumption that the physical stimulation of copulation is the principal factor involved in inducing ovulation in camelids [7]. For the purposes of the present study, the abbreviation OIF/NGF will be used to indicate NGF of seminal plasma origin.

Beta NGF is a homodimer with a molecular mass of 26–28 KDa [8], and was discovered in abundance in mouse sarcomas [9], snake venom [10], and mouse salivary glands [11]. The effects of NGF were initially thought to be restricted to nerve function and development, as indicated by a potent stimulatory effect on dorsal root ganglia during embryonic limb development in chicks [12]. More recently, NGF has been shown to play a role in a variety of non-neuronal systems such as in immune-related [13], inflammatory [14], reproductive [15], and endothelial tissues [16]. The biological actions of NGF are mediated by interaction with two receptors. Tyrosine kinase A (trkA) is a high affinity receptor for NGF and mediates its neurogenic effects (e.g., survival of dorsal root ganglia neurons in mice [17], or induction of neurite outgrowth in PC_12_ cells in vitro [18]). A non-specific low-affinity receptor (p75NTR) has been implicated in mediating trkA activation, increasing the affinity of trkA for NGF, and inducing apoptosis in cell culture [19]. The p75NTR receptor also has a low affinity interaction with other neurotrophins such as brain-derived neurotrophin factor and neurotrophin 3 [20].

Nerve growth factor has been implicated as a local mediator at different stages of development of the reproductive system. In the infantile NGF knock-out mouse, primary and secondary follicle populations were lower than in the wild type mouse [21], suggesting that the NGF signaling system has a role in fetal ovarian development. In prepubertal rats in which ovarian superstimulation was induced with equine chorionic gonadotropin, administration of anti-NGF or a trkA blocker into the ovarian bursa on the expected day of the LH surge impaired ovarian prostaglandin E2 production and reduced the ovulatory response [22]. In addition, a role in the maintenance of follicular and luteal vasculature was reflected in vascular cell proliferation of neonatal rat ovaries cultured in vitro after treatment with NGF, either directly or through synthesis of vascular endothelial growth factor [23].

A novel endocrine effect of OIF/NGF was discovered in a series of studies on ovulation in species categorized as induced ovulators (reviewed in [24]). Intramuscular administration of seminal plasma (containing OIF/NGF) in llamas and alpacas elicited a surge in plasma LH concentrations, followed by ovulation in >90 % of animals, and was associated with enhanced CL development [2]. It was concluded that the mechanism involves a central effect on the hypothalamus or pituitary gland via a systemic route [25]. However, the results of later studies in cattle (a spontaneous ovulator) provided rationale for the hypothesis that the luteotrophic effect of OIF/NGF is mediated by a local route. Although treatment with purified OIF/NGF did not induce ovulation in pre-pubertal heifers, treatment during the first follicular wave in post-pubertal heifers hastened the emergence of the following follicular wave and was luteotrophic [26]. Similarly, in a subsequent study in cattle, the administration of bull seminal plasma (containing 250 μg of OIF/NGF) did not elicit an LH response or ovulation, but did enhance CL development [27]. Plasma progesterone concentrations increased more rapidly and the CL lifespan was longer in the seminal plasma-treated group than in the control group. Surprisingly, ovulation occurred more synchronously in the seminal plasma-treated group (within a period of 4 h) than in the control group (within a period of 18 h [27]). The mechanisms by which OIF/NGF induced the ovarian changes in cattle are unknown. Although NGF and its receptors have been detected in bovine and porcine ovaries [28], their temporal expression in the ovary in relation to follicular dynamics, ovulation, and CL development have not been characterized.

To determine the role of OIF/NGF at the level of the ovary, the objective of the present study was to characterize the spatial and temporal distribution of trkA in ovarian follicles and CL at known stages of the estrous cycle, and to test the hypothesis that the luteotrophic effect of OIF/NGF is mediated by an increase in trkA receptors in the ovulatory follicle and early CL.

## Methods

### Animals

Non-lactating Hereford-cross cows (*n* = 6) and sexually mature heifers (*n* = 8) from the research herd at the University of Saskatchewan Goodale Farm were used from August to October. The experimental protocol was approved by the University Committee on Animal Care and Supply and conducted in accordance with the guidelines of the Canadian Council on Animal Care.

### Experimental design

**Fig. 1:**
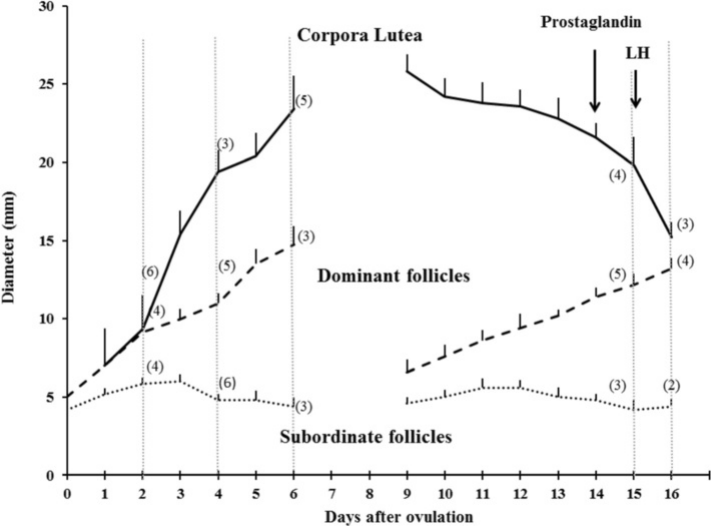
Experimental design showing follicle and CL diameter profiles (mean ± SEM) in cattle preceding unilateral ovariectomy (*vertical dashed lines*) on Days 2, 4, 6, and the pre-ovulatory period just before and just after treatment with LH. The number in parentheses accompanying the *vertical dashed lines* reflects the number of the respective structures (i.e. follicles, CL) analyzed per time point. For illustration purposes, dominant follicle diameters of the second wave were normalized to the mean day of emergence of Wave 2

The ovaries were examined daily by transrectal ultrasonography to detect ovulation (Day 0). Animals were then assigned randomly in replicate to be unilaterally ovariectomized on Day 2, 4, 6, or in the pre-ovulatory period either just before, or just after, the LH surge. Animals assigned to the pre-ovulatory groups were given a luteolytic dose of prostaglandin F_2α_ (500 μg cloprostenol im, Estroplan, Vétoquinol, Georges Lavaltrie, QC, Canada) during the luteal phase when the dominant follicle of the second follicular wave was ≥10 mm and growing. Animals assigned to the Pre-LH group were ovariectomized 24 h after prostaglandin treatment. Animals assigned to the Post-LH group were given pLH (25 mg Luthropin im, Bioniche, Belleville, Ontario, Canada) 24 h after prostaglandin treatment and were ovariectomized 18 h later. After one complete interovulatory interval following the first ovariectomy, animals were re-assigned randomly to a different day-group on which the remaining ovary was removed (*n* = 2 to 5 ovaries per day-group; Fig. 1).

### Ultrasonographic monitoring

The ovaries were examined daily by transrectal ultrasonography using a 7.5 MHz linear-array probe (Mylab 5, Esaote North America Inc., Indianapolis, Indiana, USA). The CL and ovarian follicles ≥4 mm were individually identified and monitored from day-to-day to determine luteal and follicular wave status. Wave emergence was defined as the day on which the follicle destined to become dominant was first detected at a diameter of 4–5 mm. If the future dominant follicle was first detected at 6 mm, the previous day was taken as wave emergence [29]. Ovulation was defined as the sudden disappearance of a follicle ≥10 mm from one examination to the next [30].

### Ovariectomy and tissue handling

Unilateral ovariectomy was performed via colpotomy in the standing position under caudal epidural anesthesia using 2 % (w/v) lidocaine HCl with 0.01 mg/ml epinephrine [31]. An incision was made in the dorsolateral aspect of the vaginal fornix and the peritoneum was manually punctured after blunt dissection through the adventitia. After manually compressing the mesovarium with a lidocaine-soaked gauze, the ovary containing the structure of interest was removed using a chain écraseur. Within a few minutes of collection, the ovarian artery was cannulated and perfused with 20 ml of cold phosphate buffered saline (PBS; pH = 7.4) followed by 20 ml of 4 % paraformaldehyde in PBS (pH = 7.4). The ovary was then immersed in the same fixative for 24 h at 4 °C. After the fixation period, ovaries were rinsed in PBS (3 times for 15 min. each), and stored in PBS at 4 °C. Cows were treated post-operatively with procaine penicillin G (20,000 IU/kg.) im daily for 3 days.

### Immunohistochemistry

The fixed ovaries were trimmed such that the structures of interest, previously identified by ultrasonography, were exposed for sectioning. The trimmed ovarian tissues were placed in plastic cassettes and dehydrated in graded ethanol solutions (50, 70, 90, 95, and 100 %), cleared in xylene, and embedded in blocks of paraffin. The tissue blocks were sectioned at a thickness of 5 μm and mounted on poly-L-lysine coated glass slides. Enzymatic antigen retrieval was performed using a concentration of 2 mg/ml of pepsin (Sigma, St. Louis, Missouri, USA) in a 0.01 N HCl solution (pH = 1.5) for 20 min at room temperature. Slides were then washed in PBS, and incubated in blocking buffer (1 % bovine serum albumin in PBS) for 1 h. Slides were incubated overnight at 4 °C with a primary antibody (rabbit anti-human trkA, Santa Cruz Biotechnologies, Santa Cruz, California, USA) diluted 1:200 in 1 % BSA in PBS. The next day, slides were washed and incubated for 2 h with a secondary antibody (goat anti-rabbit IgG, Alexa 488, Life Technologies, Burlington, Ontario, Canada) diluted 1:400 in 1 % BSA in PBS. After washing, slides were counter-stained with DAPI, cover-slipped, and stored (≤1 week) for examination by confocal fluorescence microscopy (Leica LSM, Wetzlar, Germany). The specificity of the antibody was tested by pre-adsorbing the primary antibody with trkA peptide for 1 h at room temperature or by omitting the primary antibody from the incubation process; both procedures prevented the detection of immunoreaction during assessment. Additionally, histologic sections from every ovary were stained with hematoxylin-eosin to assess and identify microscopic details, as described previously [32].

### Image analysis

Confocal fluorescence images of the follicular wall and the CL were analyzed with ImageJ software (NIH, Bethesda, Maryland, USA). The proportion of positive cells, the intensity of the immuno-reaction, the area stained, and the intracellular distribution of the immuno-reaction were estimated in the granulosa and theca layers of dominant and subordinate follicles. At least two images per follicle were obtained and analyzed. Each image contained information corresponding to trkA immuno-reactivity (green channel, Alexa 488) and the nuclear counterstain (blue channel, DAPI). The follicular basement membrane was used to differentiate between granulosa and theca layers, and was manually outlined using the aid of nuclear morphology (cells in the granulosa and theca display different nuclear shape). The theca interna was defined as the region extending 100 μm from the basement membrane into the ovarian stroma. The granulosa layer was defined as the area from the basement membrane into the follicular lumen. The follicular wall was considered a composite of the granulosa layer and theca interna. The proportion of cells that were immuno-positive was estimated from the total number of cells of the follicle wall, granulosa layer, or theca interna. The intensity of the immuno-reaction was estimated by creating a mask of the green channel (trkA reactive) using an algorithm to select immuno-reactive areas, and from those areas, the grayscale value per stained area was calculated. The immuno-reactive area (μm^2^) was calculated by creating a binary image (black = 0; white =256) of the green channel using a common threshold for all images; the immuno-reactive area was expressed as a percentage of the total area of the follicular wall, the granulosa layer or the theca interna. For the CL, the number of cells that were immuno-reactive was estimated from the total number of cells counted per high-powered field (63×). The intensity of the immuno-reaction of CL was analyzed, as described above. Based on the degree of granularity, two patterns of intracellular distribution of trkA were apparent; diffuse or focal. A grid overlay was placed on each image of the follicles and CL. The cells counted and classified for granularity were those in which the nucleus was overlain by the intersection of orthogonal grid lines.

### Data analysis

Differences between follicle type and day-groups were compared by two-way analysis of variance. For the CL, a day-group effect was analyzed by one-way analysis of variance. When significant differences were detected, multiple comparisons were made using the method of least significant difference. Data are presented as the mean ± SEM, and significance was considered when *P* ≤ 0.05. Cell counts from corpora lutea were pooled into early stage (Day 2 and Day 4) and later stage day-groups (Day 6, Pre-LH and Post-LH) and compared by t-test for unequal variance [33]. The intracellular distribution of trkA immuno-reactivity of dominant and subordinate follicles is expressed as mean ± SEM of the diffuse:focal ratio, and was compared as described for follicles above.

## Results

The diameters of dominant and subordinate ovarian follicles and the CL at the time of ovariectomy are shown in Fig. 1 (*n* = 2 to 5 structures/per day-group). The diameter profile of the subordinate follicle in day-groups 2, 4 and 6 is that of the largest subordinate in the excised ovary, but not necessarily the largest subordinate of the follicular wave.

**Fig. 2:**
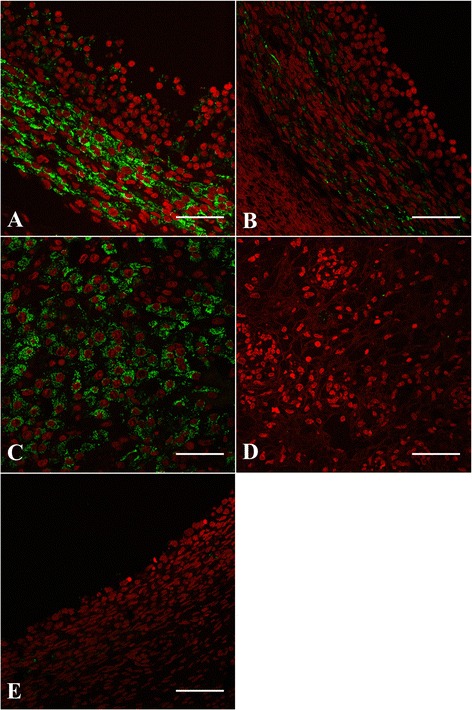
Immunofluorescence staining pattern of trkA (*green*) in a dominant follicle (**a**), subordinate follicle (**b**), CL (**c**) CL from the previous cycle (**d**), and regressing dominant follicle from the previous cycle (**e**) in cattle. *Red*: pseudo-color for nuclear counterstain. FL: Follicular lumen, GL: Granulosa layer, TL: Theca layer, FW: Follicular wall, LC: Luteal cell. Scale bar = 50 μm

The fluorescence signal in ovarian tissues (Fig. 2) was restricted to follicles and the CL. No reaction was detected in stromal cells or blood vessels, and no signal was detected in regressing follicles or the regressing CL from the previous cycle (Fig. 2d). Immuno-reactivity was also detected in the theca layer of small antral follicles ≤ 1 mm (i.e., those not detected by ultrasonography). At the cellular level, immuno-reactivity was restricted to the cytoplasm; nuclear staining was not observed. No statistical difference was detected in trkA immuno-reactivity of the dominant follicles collected after the first versus second unilateral ovariectomy; hence, data for all structures were analyzed regardless of whether collection was at the first or second ovariectomy.

### Follicles

**Fig. 3:**
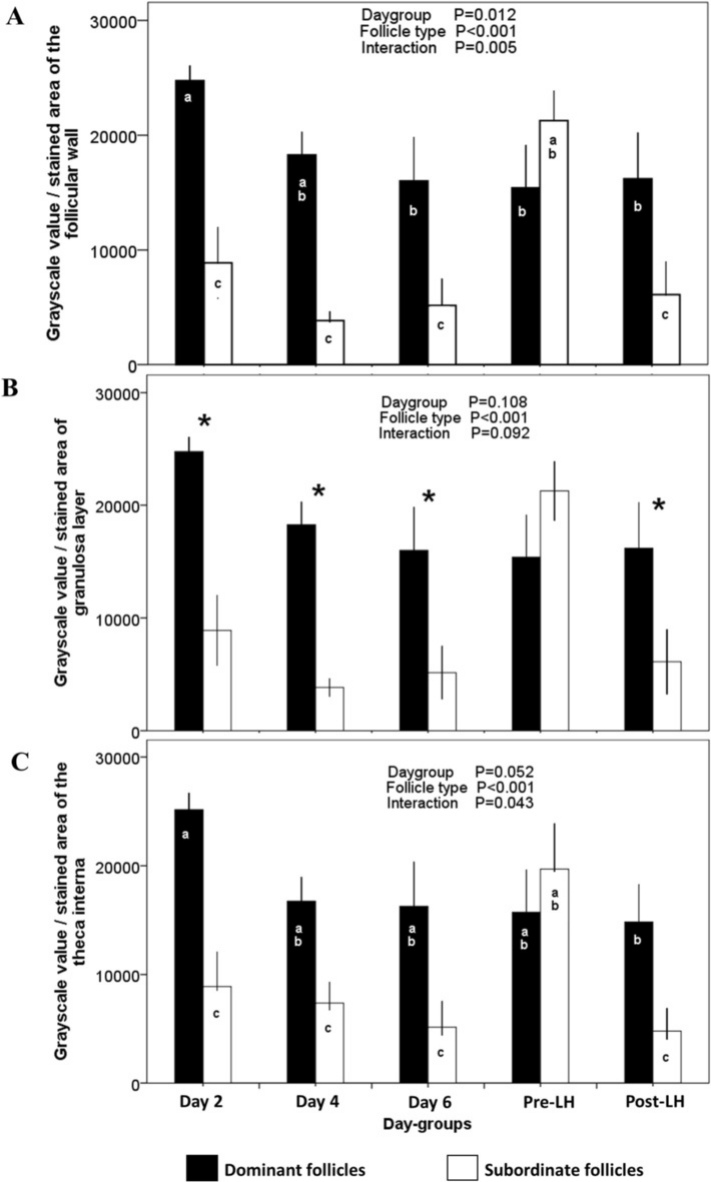
Grayscale intensity of pixels (mean ± SEM) of the trkA immuno-positive area of dominant (*black bars*) and subordinate ovarian follicles (*white bars*) collected at the time of ovariectomy (Day-groups; Day 0 = ovulation) in cattle. Intensity values (0 = *black*; 65536 = *white*) of the follicular wall (**a**), the granulosa layer (**b**), and the theca interna (**c**). ^abc^ Values with no common superscript are different (*P* < 0.05). *Difference between dominant and subordinate follicles (*P* < 0.05)

The intensity of trkA immuno-reactivity of the follicular wall was greater in dominant versus subordinate follicles in all day-groups except one, the Pre-LH group (follicle type by day-group interaction, *P* = 0.005; Fig. 3a). Among dominant follicles, the intensity of the immuno-reaction in the follicular wall was greater on Day 2 than on Day 6, Pre-LH, or Post-LH (*P* = 0.04, *P* = 0.01, *P* = 0.03, respectively). The intensity of trkA immuno-reactivity of the follicular wall of subordinate follicles remained constant among day-groups, except for the Pre-LH group in which it was similar to that of dominant follicles (Fig. 3a). A similar pattern was observed when the intensity of the immuno-reaction was analyzed with respect to the granulosa layer (Fig. 3b) or the theca interna (Fig. 3c), separately.

**Fig. 4:**
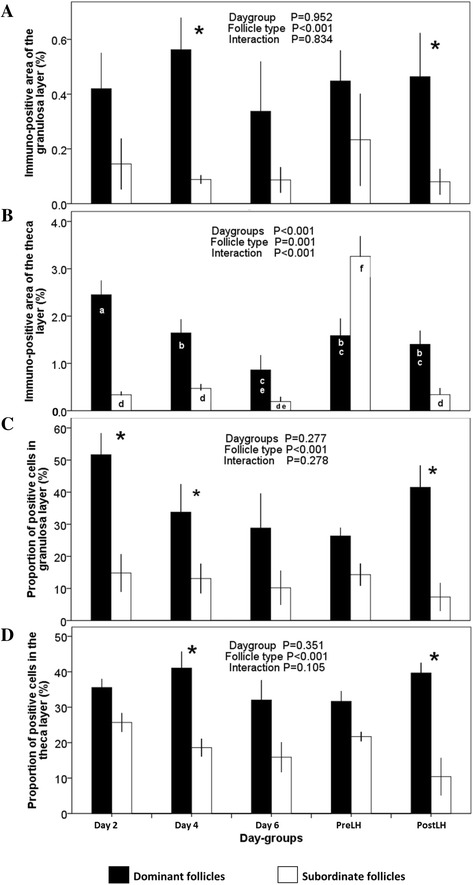
Proportion of trkA immuno-positive area in the follicular wall (granulosa and theca) of dominant (*black bars*) and subordinate follicles (*white bars*) collected at the time of ovariectomy (Day-groups; Day 0 = ovulation; mean ± SEM) in cattle. **a** Immuno-positive area of the granulosa layer (% of the total area of the granulosa). **b** Immuno-positive area of the theca layer (% of the total area of the theca). **c** Proportion of cells in the granulosa layer that were immuno-positive. d Proportion of cells in the theca layer that were immuno-positive. ^abc^ Values with no common superscripts are different (*P* < 0.05).*Difference between dominant and subordinate follicles (*P* < 0.05)

The immuno-positive area of the follicle wall was greater in dominant follicles than in subordinate follicles (*P* < 0.001), with no effect of day-group or interaction (Fig. 4a). An interaction between day-group and follicle type (*P* < 0.001) in the immuno-positive area of the theca layer was attributed to a dramatic increase in subordinate follicles of the Pre-LH group compared to other groups (Fig. 4b). A progressive reduction in the immuno-positive area of the theca layer was detected in dominant follicles from Day 2 to Day 6 (*P* < 0.05; Fig. 4b).

**Fig. 5:**
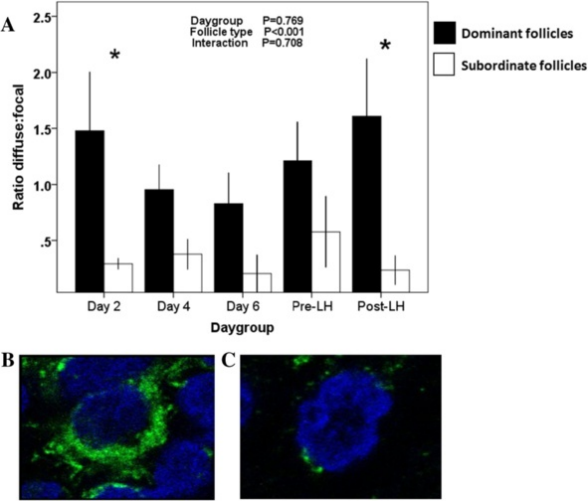
Patterns of intracellular distribution of trkA receptor in cells of the granulosa and theca layers of dominant and subordinate follicles in cattle, assessed by confocal microscopy. **a** The pattern of granularity is expressed as the ratio of diffuse versus focal distribution in dominant (*black bars*) and subordinate follicles (*white bars*) among day-groups. **b** Diffuse granularity. **c** Focal granularity. *Difference between dominant and subordinate follicles (*P* < 0.05)

The proportion of cells that were immuno-positive was greater in dominant follicles than subordinates follicles in both the granulosa (*P* < 0.001) and theca (*P* < 0.001) layers, but no effect of day-group or interaction was detected (Fig. 4c, d). The follicular wall of dominant follicles displayed a greater diffuse to focal ratio of trkA immuno-reactivity than that of subordinate follicles (*P* < 0.001), and were maximal in the Day 2 and Post-LH groups (Fig. 5).

### Corpus luteum

**Fig. 6:**
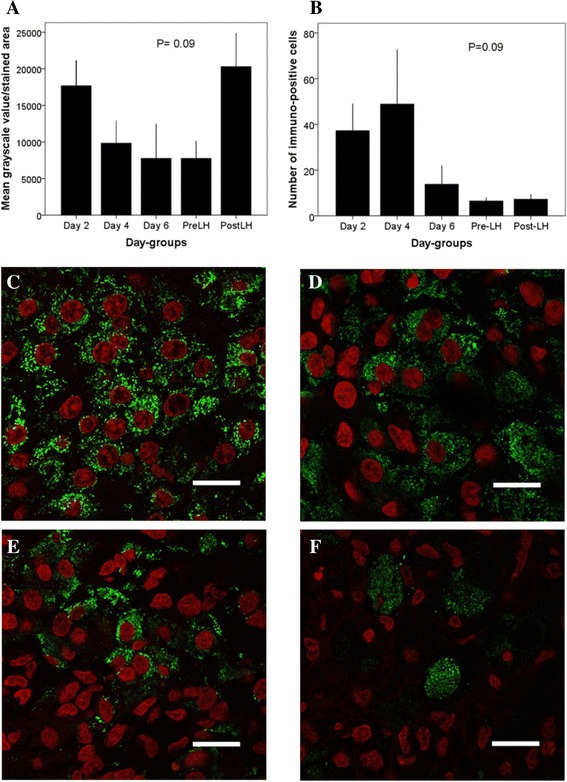
Anti-trkA staining pattern of the bovine CL collected in the periovulatory period, assessed by confocal microscopy. **a** Grayscale intensity values of immuno-reactive cells and (**b**) number of immuno-positive cells per high-powered field (mean ± SEM) in the CL among different day-groups (*n* = 3–5 ovaries per group; Day 0 = ovulation). **c**-**f** Photomicrographs depicting anti-trkA immuno-fluorescence (*green*) in bovine CL on Day 2 (**c**), Day 4 (**d**), Day 6 (**e**) and Post-LH (**f**). Cell nuclei are shown in *red* (pseudo-color). Scale = 20 μm

In luteal cells, trkA immuno-fluorescent granules were distributed homogenously within the cytoplasm of immuno-positive cells in all day-groups, but the grayscale intensity values and number of immuno-positive cells tended to differ among day-groups (*P* = 0.09; Fig. 6). In a retrospective comparison, the number of immuno-positive cells was greater in early developing CL (Days 2 and 4 combined) than in mature or regressing stage CL (Day 6, Pre- and Post-LH combined; 41.1 ± 10.4 vs 9.7 ± 3.4 cells per high-powered field; *P* = 0.01).

## Discussion

The bovine model was used in the present study as a species representative of spontaneous ovulators and because of the ability to monitor ovarian events over time in relation to putative factors controlling ovarian function [34, 35]. Antral follicular dynamics during the estrous cycle in cattle and other species is a highly coordinated phenomenon characterized by two or more waves of follicle development. Each follicular wave consists of simultaneous growth of 8 to 40 follicles, detected initially at a diameter of ≥1 mm, one of which continues to grow (dominant follicle) while the others regress (subordinate follicles) [35–37]. During the luteal phase (i.e., elevated progesterone and low LH), the dominant follicle ultimately ceases growth and begins to regress, whereas a dominant follicle during luteolysis or in the absence of a CL (absence of the inhibitory effect of progesterone on LH release) will ovulate.

Results of the present study reveal abundant expression of the NGF-specific receptor, trkA, in antral ovarian follicles and the CL throughout the estrous cycle in cattle. TrkA has been isolated in theca and granulosa cells of bovine ovarian follicles of varying sizes in ovaries collected from the abattoir [38] and in immuno-histochemical studies [28], but the physiologic role of the NGF/trkA system in ovarian function in cattle was not examined. In the present study, the granulosa and theca layers of the dominant follicle of both anovulatory and ovulatory follicular waves of the estrous cycle expressed higher levels (intensity, area stained, and proportion of positive cells) of trkA receptors than that of subordinate follicles, suggesting a role of OIF/NGF during follicle selection and maturation. Results are consistent with those of a study involving in vitro culture of isolated ovarian follicles from sheep in which concentrations of NGF in the follicular fluid were greater in follicles ≥4 mm than in those ≤3 mm [39]. Interestingly, the difference in trkA expression between dominant and subordinate follicles was most obvious during the early stage of CL development (Days 2 and 4) and after luteolysis (Post-LH); i.e., during periods of low progesterone and elevated LH pulse-frequency [40, 41]. The temporal relationship suggests that trkA receptors may be induced by LH. In this regard, NGF and trkA were detected only within 4 h before the first preovulatory LH surge at the time of puberty in rats [22].

Analysis of the CL revealed a tendency for a difference among day-groups in the number of immuno-positive cells. The difference was attributed to a greater number of immuno-positive cells during the early luteal phase (Days 2 and 4) than during mature and regressing phases (Day 6, Pre- and Post-LH). A greater number of trkA-responsive cells is consistent with the findings of a luteotrophic effect of OIF/NGF reported in cattle [26, 27]. Perhaps trkA expression in the early CL is a carry-over of trkA immuno-positive cells of the theca and granulosa layers of the preovulatory follicle, since trkA and NGF have been implicated as regulators of cyto-differentiation at follicle rupture [42]. However, in gilts trkA and NGF were detected by immunofluorescence and western blot in the CL from Day 3 to Day 16 of the estrous cycle [43], indicating a potential role of the NGF/trkA system during the lifespan of the CL.

An unexpected finding in our study was the high expression (intensity and area stained) of trkA receptors in subordinate follicles of the Pre-LH group. An earlier histomorphometric study of bovine follicular populations [32] described a thickening or hypertrophy (luteinization) of the theca interna of subordinate follicles in the static and early regressing phases in both ovulatory and non-ovulatory waves. In the present study, we found a greater intensity and area of trkA immunoreaction in the subordinate follicles in the Pre-LH group but not in the Day 6 group. Perhaps the increase in trkA receptors and luteinization of the wall of subordinate follicles is a consequence of an increase in LH pulse frequency subsequent to luteolysis [41, 44]. The relative absence of trkA immuno-reactivity in the subordinate follicles of the Post-LH group may be attributed to a more advanced state of atresia than in the Pre-LH group.

In addition, we found that early antral follicles (<1 mm in diameter) were immuno-reactive to trkA, similar to that previously reported in cattle [28], reinforcing the idea that OIF/NGF is involved in antral follicle growth and maturation. In this regard, follicular wave emergence was hastened in cows treated with OIF 6 days after ovulation [26]. Taken together, the pattern of expression of trkA in the bovine ovary suggests that OIF/NGF has effects not only in the final stages of follicle development (i.e., preovulatory follicle), but also at earlier stages of folliculogenesis, as reported in mice [21].

The trkA receptor has been shown to be translocated from docking sites to the plasma membrane during NGF-induced differentiation in PC12 cells [45]. In the present study, the difference in the intracellular distribution of trkA immuno-reactivity between dominant and subordinate follicles was maximal in the Day 2 and the Post-LH groups. Diffuse immuno-reactivity seen in the present study was interpreted as a wide and active distribution of trkA receptors within the cell. Conversely, a focal intracellular distribution was interpreted as an accumulation of inactive receptor in a specific cytoplasmic compartment. Interestingly, the difference in the diffuse:focal distribution between dominant and subordinate follicles was greatest when progesterone concentrations were at expected minima [40, 46], suggesting that intracellular trafficking of trkA receptor in the ovarian follicle is affected directly by ambient progesterone or indirectly by changes in LH pulse-frequency.

## Conclusion

In conclusion, data support the hypothesis that the luteotrophic effect of OIF/NGF in cattle is mediated, in whole or in part, by a rise in trkA receptor expression in the ovulatory follicle and early CL. That is, the follicular and luteogenic effects of OIF/NGF in cattle (a spontaneous ovulator) are mediated directly at the level of the ovaries through interaction with trkA on theca and granulosa cells, rather than through gonadotropin release by interaction with trkA receptors in the hypothalamus and pituitary gland. Distinct differences in trkA expression between dominant and subordinate follicles, particularly when circulating progesterone is minimal (during early luteal development and after luteolysis), are consistent with a role of OIF/NGF in follicle selection and early luteogenesis.

## Abbreviations

BSA, bovine serum albumin; CL, corpus luteum; HCl, hydrochloric acid; LH, luteinizing hormone; NGF, nerve growth factor; OIF/NGF, ovulation-inducing factor/nerve growth factor; PBS, phosphate buffer saline; TrkA, tyrosine kinase A
